# Supplementary material for: Weighing up the evidence used by direct-to-consumer stem cell businesses
Source: Stem Cell Reports. 2021 Nov 11;16(12):2852–60. doi: 10.1016/j.stemcr.2021.10.007 (PMC8693621; doi:10.1016/j.stemcr.2021.10.007)
Supplement: Document S1. Supplemental experimental procedures and Tables S2, S3, and S5 [file mmc1.pdf]

Stem Cell Reports, Volume *16*

Supplemental Information

Weighing up the evidence used by direct-to-consumer stem cell businesses

Margaret Cook, Alexandra Richey, David A. Brafman, and Emma K. Frow

Supplemental Information

Table S1. Data summary for direct-to-consumer stem cell businesses in the Southwest US (related to Experimental Procedures). State locations are provided for each business, but specific business names are not identified.

See Excel file for Table S1.

Table S2. List of all medical boards cited by care providers across the businesses characterized (*n*=179 care providers across 59 businesses), grouped by medical specialty. Boards represented by the American Board of Medical Specialties (ABMS) and the American Osteopathic Association (AOA) are noted. Related to **Fig. 3**.

**Categories**	**Medical Boards Contained in Category**	**ABMS or AOA**
Cosmetic	American Board of Plastic Surgery	ABMS
	American Board of Cosmetic Surgery	
	American Board of Facial Plastic and Reconstructive Surgery	
	American Academy of Aesthetic Medicine	
	American Board of Hair Restoration Surgery	
	American Board of Dermatology	ABMS
General	American Board of Family Medicine	ABMS
	The American Board of Internal Medicine	ABMS
	American Board of Preventive Medicine in Occupational Medicine	ABMS
	National Commission on Certification of Physician Assistants	
	American Academy of Nurse Practitioners	
	American Association of Medical Assistants	
Pain Medicine	American Board of Pain Medicine	
	American Board of Interventional Pain Physicians	
Alternative	American Osteopathic Academy of Sports Medicine	
	American Osteopathic Board of Physical Medicine and Rehabilitation	ABMS
	American Board of Holistic Medicine	
	National Board of Osteopathic Medical Examiners	
	American Osteopathic Board of Family Practice	AOA
	American Board of Functional Medicine	
	American Osteopathic Board of Family Physicians	AOA
	American Osteopathic Board of Neuromusculoskeletal Medicine	AOA
	National Certification Commission for Acupuncture and Oriental Medicine	
Orthopedics	American Board of Orthopaedic Surgery	ABMS
	American Board of Spine Surgery	

Sports Medicine & Rehab	American Board of Physical Medicine and Rehabilitation	ABMS
Surgery	American Board of Anesthesiology	ABMS
Regenerative Medicine	American Board of Anti-Aging & Regenerative Medicine	
Other	The American Board of Otolaryngology	ABMS
	American Board of Psychiatry and Neurology	ABMS
	American Board of Radiology	ABMS
	American Board of Urology	ABMS
	American College of Thermography	
	American Board of Addiction Medicine	

Table S3. Professional associations listed by all care providers on the business websites studied (*n*=179 care providers across 59 businesses). Professional associations were grouped into nine categories; a complete list of associations is available in Table S4.

**Medical Specialty**	**Professional associations at sole focus businesses (# of care providers)**	**Professional associations at main focus businsses (# of care providers)**	**Number of professional associations represented**
Alternative Medicine	2	14	13
Cosmetic	10	4	9
General	20	4	19
Orthopedics	9	15	9
Pain Medicine	1	8	6
Sports Medicine & Rehab	0	5	3
Regenerative Medicine	4	3	2
Surgery	8	5	7
Other	8	7	14
**Total Number of Professional Associations**	**76**	**65**	**82**
Number of Doctors listing Professional Associations	25	25	
Total Number of Doctors	71	108	

Table S4. List of all professional associations cited by care providers across the businesses characterized, grouped by medical specialty as in Table S3.

See Excel file for Table S4.

Table S5. Type of statement made in invoking the FDA, broken down by sole-focus (*n*=27) and main-focus clinics (*n*=32). Specific quotations illustrate the types of statements classified as "explicitly" acknowledging the unregulated status of SCBIs (blue) or "ambiguous" with respect to the regulatory status of SCBIs (yellow). Color-coding matches the pie charts in **Fig. 4B**.

Categorization of FDA Statement	Total # of businesses	# of sole-focus businesses	# of main-focus businesses	Example statement
Explicit statement that stem cell treatments are not FDA-approved	**13**	8	5	"Regenerative medicine (RM) is a fast-growing, highly-developed technology that is increasingly being used to manage conditions such as chronic and acute pain. The FDA has not yet approved the use of regenerative treatments and medicine to remedy every pain condition and case. However, regenerative treatments and medicine might be appropriate for you, depending on your specific case."
Explicit statement that stem cell treatments are not FDA-approved but suggesting compliance with 21 CFR Part 1271	**7**	7	0	Is our procedure FDA-approved? "NO. However, [our] surgical procedures fall under the category of physician's practice of medicine, wherein the physician and patient are free to consider their chosen course of treatment. The FDA does have guidelines about treatment and manipulation of a patient's own tissues. At [company] we meet these guidelines by providing same day treatment with the patient's own cells that undergo no manipulation and are inserted during the same procedure."
Explicit statement that stem cell treatments are not FDA-approved but suggesting these treatments do not require FDA approval/clearance	**1**	1	0	"This procedure is not FDA approved and cannot be FDA approved because it does not involve anything other than a person's own tissue. The FDA does not approve or disapprove of any procedures like this, because it is a type of operation that they have no domain over. Instead, this is a procedure in which the person's own cells are transferred in a concentrated form into an area where he or she may need them."
Statement claiming compliance with the FDA regulations set forth in 21 C.F.R. Part 1271 while also suggesting that stem cell treatments do not require FDA approval/clearance	**2**	1	1	"This is a medical procedure; the FDA does not "approve" or "disapprove" medical procedures (like gall bladder surgery for example). Medical procedures are performed at the discretion of the practicing physician and their state's board. All of [Company's] Stem Cell and Blood Platelet Procedures performed in the U.S. today are same-day procedures that are compliant with CFR 21 Part 1271, falling under the same surgery exemption discussed in 1271.15 (b)."
Statement invoking an FDA-registered clinic/facility	**2**	2	0	"We work with [other company], an FDA-approved stem cell provider. Every sample we receive is microbiology tested and registered with the FDA, Tissue Bank and NIH & FWA."

Statement invoking an FDA-registered device	**1**	0	1	"Our medical director and founding surgeon, [Doctor], offers our hair loss patients the most sophisticated FDA approved stem cell device for obtaining adult stem cells from the fat cells of patients."
Suggesting broad compliance with FDA	**5**	0	5	"[Business] follows all FDA guidance related to stem cell therapy. You are encouraged to consult with your primary care physician prior to undergoing a stem cell therapy."
Statement claiming that stem cell treatments are FDA approved/cleared	**2**	0	2	"[Business] offers the only FDA approved autologous adipose stem cell regeneration system. The potential and future of the use of stem cells in aiding recovery of multiple soft tissue injuries is now offered in [region]!"
Do not mention the FDA	**25**	8	18	N/A

Supplemental Experimental Procedures

Online data collection

DTC stem cell businesses were characterized between late July and early September 2020. Information was collected from the public sections of business websites and collated in an Excel spreadsheet. For each characterized business, screenshots were taken of each webpage presenting information that could be interpreted as evidence supporting the SCBIs on offer at the clinic (using Snagit software), to construct an archive for future reference. The data collected reflects the public presentation of a stem cell business; it is assumed to be accurate, but is not guaranteed to represent the actual practices of a given business. For example, a given clinic might not list all the conditions they treat using SCBIs, or might not in practice treat all the conditions they list online.

Peer-reviewed scientific papers. After identifying the subset of clinics citing peer-reviewed scientific papers, citations for each article were obtained and the corresponding articles found through PubMed. A total of 261 journal articles were identified. The abstract for each paper was read, and a set of categories was iteratively developed by the authors (A.R. and E.K.F.) to evaluate each paper in relation to the SCBIs on offer at the business citing the paper. The following categories were used: (1) whether the paper was investigating a specific stem-cell-based treatment, (2) what condition was being tested or treated using stem cells, (3) whether the research subjects were humans or animals, (4) the sources of stem cells used in the paper, (5) whether the cells in the preparation had been cultured or manipulated, (6) the size of the study performed, and (7) whether or not the study showed that stem cell treatment resulted in positive clinical outcomes compared to the control group. This information was cross-referenced with the medical conditions treated by the business citing the paper, together with the treatment method(s) presented by the business and the source(s) of stem cells they claimed to use. Based on this information, a determination was made for each cited paper about whether it supported the SCBIs offered by that business.

Among the businesses evaluated, three larger franchises were represented: Regenexx, R3 and the Cell Surgical Network (CSN). CSN and Regenexx have both published research papers by their lead physicians and researchers; these were analyzed as described above. R3 also referenced peer-reviewed articles in their informational videos, but these were not authored by individuals affiliated with R3. These were also analyzed as described above. CSN also hosts a large database of regenerative medicine research on their website. The purpose of this database is stated as

giving physicians and researchers "the opportunity to share not only their data, but their vital clinical experiences [in order to help] each other to achieve higher levels of scientific understanding and [optimize] medical care."[1] This database covers many forms of regenerative medicine and has entries discussing research for over 30 medical conditions in the fields of urology, cardiology and pulmonology, orthopedics, neurology, autoimmune diseases, and ophthalmology. The entries in the database include peer-reviewed journal articles covering stem-cell-based and non-stem-cell-based treatments, review articles, and information on ongoing clinical trials. Since this extensive database is stated as being primarily for the benefit of physicians and researchers (not patients considering SCBIs), these entries were not individually evaluated.

Medical Specialties. The care providers listed for each stem cell business were copied into the data collection spreadsheets, together with any specialty and/or board certification information listed on the business website (**Supplemental Table S2**), and any professional associations or memberships listed (**Supplemental Table S4**). Once identified, board certifications and professional affiliations were grouped into 9 categories for analysis (**Fig. 3B** and **Supplemental Table S3**).

Rate of clinic turnover. Since the publication of Frow et al (2019), we note that approximately 33% of the sole-focus businesses (14 out of 42) no longer had a web presence or no longer offered SCBIs, and that a similar proportion of the main-focus businesses (24 out of 65) no longer had a web presence or no longer offered stem-cell-based or regenerative medicine interventions. This is higher than the 10% turnover noted by Wu et al. (2019) in their study of stem cell clinics in California, Florida and Texas between 2016-2018. These numbers suggest a highly dynamic market for SCBIs. We speculate that the higher turnover might reflect the formalization of FDA guidance regarding cell- and tissue-based products in November 2017, with the grace period for compliance to expire in May 2021. Some businesses had also shifted their emphasis with respect to SCBIs since Frow et al. (2019), with 6 out of 42 businesses moving away from a sole focus on SCBIs to offering a greater variety of services. Conversely, 6 out of 65 of the 'main-focus' businesses previously characterized had narrowed their focus exclusively to SCBIs.

---

[1] See https://stemcellrevolution.com/about-us/our-mission/.

References

Fu, W., Smith, C., Turner, L., Fojtik, J., Pacyna, J.E., and Master, Z. (2019) Characteristics and scope of training of clinicians participating in the US direct-to-consumer market for unproven stem cell interventions. JAMA 321(24), 2463-2463.
