# Supplementary material for: Weighing up the evidence used by direct-to-consumer stem cell businesses
Source: Stem Cell Reports. 2021 Nov 11;16(12):2852–60. doi: 10.1016/j.stemcr.2021.10.007 (PMC8693621; doi:10.1016/j.stemcr.2021.10.007)
Supplement: Document S2. Article plus supplemental Information [file mmc3.pdf]

Stem Cell Reports
Report
OPEN ACCESS

Weighing up the evidence used by direct-to-consumer stem cell businesses

Margaret Cook,[1] Alexandra Richey,[1] David A. Brafman,[1] and Emma K. Frow[1,2,*]
[1]School of Biological & Health Systems Engineering, Arizona State University, Tempe, AZ 85287, USA
[2]School for the Future of Innovation in Society, Arizona State University, Tempe, AZ 85287, USA
*Correspondence: emma.frow@asu.edu
https://doi.org/10.1016/j.stemcr.2021.10.007

SUMMARY

Hundreds of businesses across the United States offer direct-to-consumer stem-cell-based interventions that have not been approved by the Food and Drug Administration. Here, we characterize the types of evidence used on the websites of 59 stem cell businesses in the Southwest United States to market their services. We identify over a dozen forms of evidence, noting that businesses are less likely to rely on "gold-standard" scientific evidence, like randomized clinical trials, and instead draw substantially on forms of evidence that we identify as being "ambiguous." Ambiguous evidence has some scientific or medical basis, but its interpretation is highly context-dependent. These findings highlight the interpretive responsibility placed on prospective patients. We identify actions for regulators and professional societies to assist with evaluating evidence, but caution that focusing on the (in)validity of particular evidence types is unlikely to eliminate demand for stem-cell-based treatments in this complex marketplace.

INTRODUCTION

There currently exist a large number of US clinics offering stem-cell-based interventions (SCBIs) for many different medical conditions. Researchers have been tracking this marketplace, publishing studies that characterize these clinics and their care providers (Frow et al., 2019; Fu et al., 2019; Knoepfler and Turner, 2018; Turner, 2018; Turner and Knoepfler, 2016). Scientific and bioethical communities continue to call for stronger regulation and oversight of this sector, emphasizing the "unproven" nature of the SCBIs routinely on offer and highlighting the lack of evidence demonstrating their clinical efficacy (ISSCR, 2015; Sipp et al., 2017; Zarzeczny et al., 2018). Yet the marketplace remains strong, suggesting that the presence of robust clinical evidence is not a key factor behind its emergence and growth. In this paper, we start from this observation to explore the variety of forms of evidence used in marketing direct-to-consumer SCBIs.

Specifically, we extend an earlier characterization of stem cell clinics in the Southwest United States (Frow et al., 2019) to present a detailed account of the different forms of evidence used on their websites. This study fits into a growing body of research focused on the marketing strategies of stem cell clinics. It complements content and discursive analyses of the marketing strategies around SCBIs, which identify specific types of appeals made in promoting regenerative medicine practices (Rachul et al., 2015; Munsie et al., 2017; Knoepfler, 2017; Erikainen et al., 2020). The forms of evidence we identify overlap substantially with the "tokens of legitimacy" identified by Sipp et al. (2017). We build on the categorization offered by Sipp et al. (2017) and the empirical account provided by Munsie et al. (2017), and here present a small sample of the frequency with which different forms of evidence are used in marketing SCBIs. We highlight which specific "tokens of legitimacy" are more- or less-routinely drawn upon, and in doing so identify priorities for potential action. Our dataset reveals the "ambiguity" of much of the evidence presented by clinics, where the form of evidence itself might have some scientific or medical standing (for example, the peer-reviewed scientific paper) but where the interpretation of this evidence is highly context-dependent. By focusing on ambiguity, we draw attention to the interpretive responsibility faced by prospective patients in navigating the SCBI marketplace, in the United States and beyond.

RESULTS

Businesses offering SCBIs use many forms of evidence in marketing their services. In characterizing 59 stem cell businesses in the Southwest United States, we identified and tabulated the frequency of 13 different forms of evidence (Figure 1), loosely ordered along a spectrum from more scientifically credible to less scientifically credible forms of evidence. Overall, few marked differences were noted between businesses focused exclusively on stem cells (sole-focus businesses), and businesses for which stem cells were a main offering but not the only kind of intervention on offer (main-focus businesses).

As indicated in Figure 1, most businesses marketing SCBIs tended to use forms of evidence that fall in the middle of the evidence spectrum. More specifically, these businesses commonly used scientific language or jargon in marketing their services, but infrequently cited or invoked what might be considered gold-standard forms of scientific

This is an open access article under the CC BY license (http://creativecommons.org/licenses/by/4.0/).

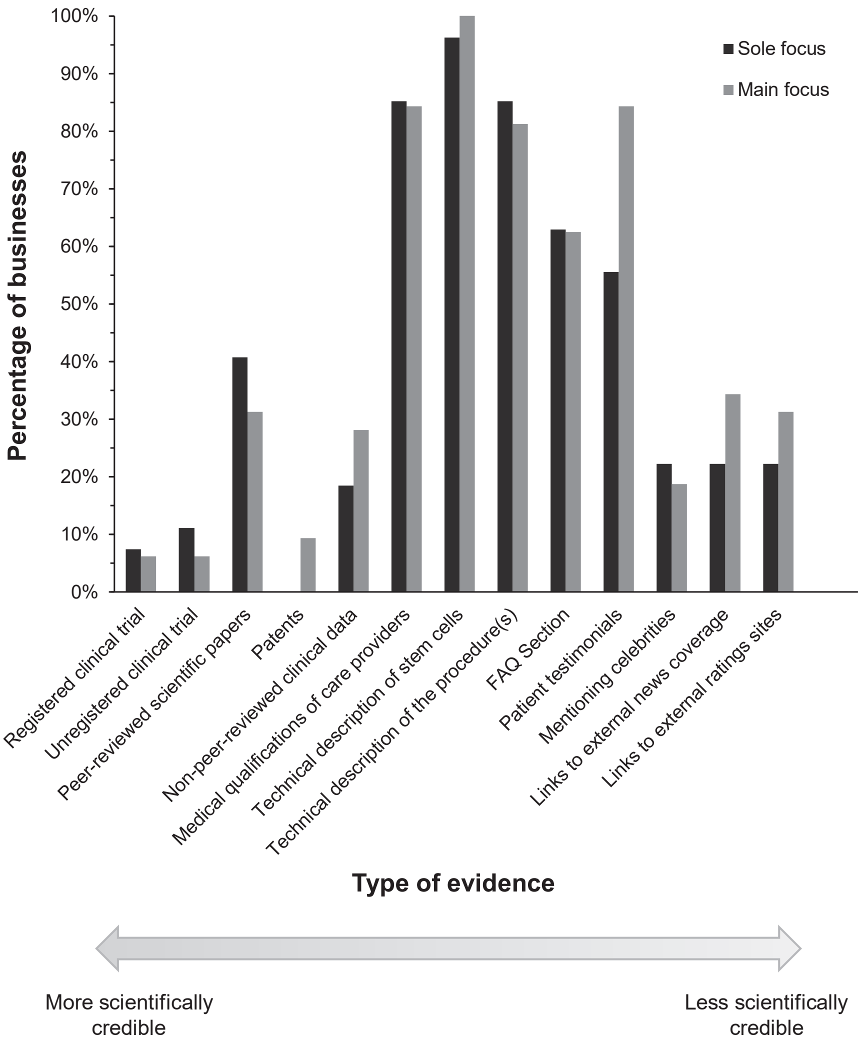

Figure 1. Forms of evidence used on stem cell business websites

Types of evidence are ordered on an approximate spectrum from more scientifically credible or accepted forms of evidence (left) to less scientifically credible (right). Individual businesses often make use of more than one type of evidence, and so may be represented multiple times (n = 59 businesses).

or clinical evidence. For example, there was almost-ubiquitous use of general descriptions of stem cells that presented authoritative, "textbook"-style information (n = 58, 98%) without providing credible references to support this information. Technical descriptions of the medical procedures on offer were present on over 80% of the business websites examined. The level of technical jargon varied among businesses, with most defining key technical terms used and presenting them in accessible ways. A common format for conveying technical information about the theory and practice of regenerative medicine was through the use of a "frequently asked questions" section (n = 37, 63%).

Clinical data

Direct-to-consumer stem cell businesses invoked clinical data in multiple ways on their websites. Registered clinical trials were mentioned by four of the 59 businesses. Three businesses mentioned being actively involved in active clinical trials, and the fourth provided a link to a published clinical trial they had not been involved with. A further five businesses mentioned clinical trials, but these had not been formally registered with clinicaltrials.gov and no results were available on the clinic websites.

A quarter of businesses (24%, n = 14) provided some non-peer-reviewed clinical data on their websites, for example mentioning the number of patients they had treated and/or referring to aggregated patient outcome data. Half of these businesses (n = 7 of 14) were associated with one of the three main direct-to-consumer SCBI franchises in the United States (Regenexx, Cell Surgical Network, and R3).

A more common approach to presenting information about clinical outcomes of SCBIs was in the form of patient testimonials (n = 42 businesses, 71%), either as written quotations or video testimonials from individuals presenting themselves as satisfied patients. These invariably described positive treatment outcomes and/or offered

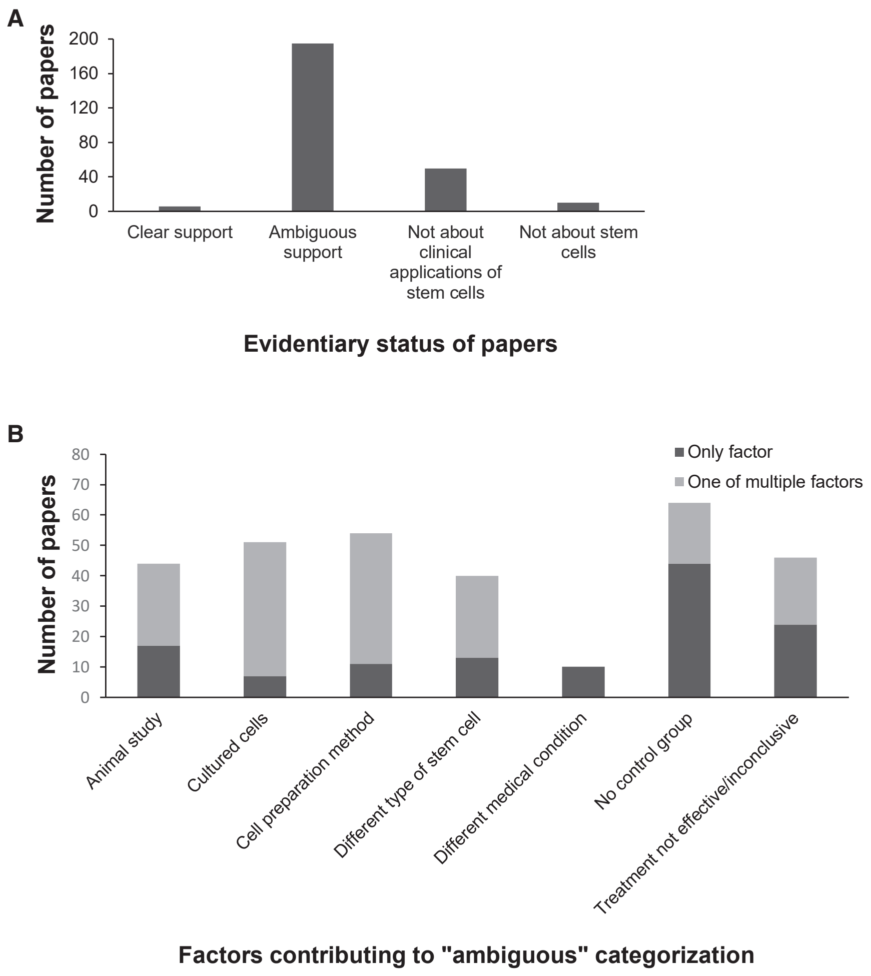

Figure 2. Peer-reviewed scientific papers

(A) Degree to which papers on clinic websites support the specific intervention(s) offered by that clinic (n = 261 papers).

(B) Specific criteria used to classify a given paper as lending "ambiguous" support to a business' services, as identified in (A) and described in the results section (n = 195 papers). An individual paper might fall into more than one category and so be represented multiple times.

glowing reports of the care provider and treatment process. A smaller number of businesses (n = 12, 20%) mentioned celebrity figures (often athletes) who had pursued SCBIs; little clinical information was typically provided in these instances, and outcomes were rarely mentioned.

Peer-reviewed scientific papers

Of the 59 businesses characterized, 21 listed specific, peer-reviewed scientific journal articles on their websites as evidence in support of SCBIs. These 21 businesses cited a total of 261 journal articles published in 170 different journals. Approximately 10% (n = 27) of the articles were published in leading stem cell and scientific journals (as listed by Knoepfler [2020]); fewer than 1% (n = 2) were published in potentially predatory journals (as defined by Beall's list, see https://beallslist.net/).

In citing research papers, about 25% of businesses (5 of 21) offered a commentary or summary of the article findings; the rest provided a citation but did not offer any interpretation of the research findings for prospective patients.

Further analysis revealed an ambiguous relationship between the research papers cited and the specific treatments on offer at the clinic citing the paper (Figure 2A). We evaluated each of the 261 journal articles with respect to the SCBIs marketed by the business citing them. Only 2% (n = 6) of the articles presented direct evidence in support of the specific treatments being offered by the clinics referencing them – that is, they described human clinical studies involving the same kind of (minimally manipulated) stem-cell-based preparation to treat the same medical condition offered by the clinic.

A further 75% of the articles cited (n = 195) did focus on clinical applications of stem cells, but the findings presented did not lend direct support for the specific interventions marketed by the business citing them. We categorized this set of papers as offering "ambiguous" evidence with respect to the services offered by the clinic (Figure 2B). The reasons for including a journal article in this category included (1) reporting on an animal rather than a human study, (2) using cultured stem cells (rather than the

A

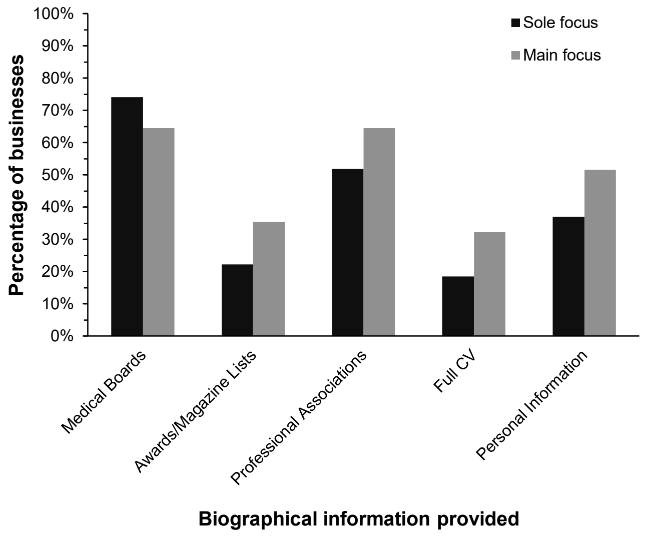

Figure 3. Medical expertise and qualifications of care providers

(A) Types of biographical information provided for at least one care provider at a given stem cell business (n = 59 businesses). Personal information includes information about a care provider's family and/or hobbies.

(B) Board certifications of all care providers listed on the business websites studied (n = 179 care providers across 59 businesses). A board certification was classified as "unknown" if board certification was mentioned on a clinic website but no specific specialty or board was listed. One care provider may have more than one board certification. Medical boards and professional affiliations were grouped into nine categories; complete lists are available in Tables S2, S3, and S4.

B

Medical Specialty	Board certifications at sole-focus businesses (# of care providers)	Board certifications at main-focus businsees (# of care providers)	Number of different medical boards represented
Alternative Medicine	3	7	9
Cosmetic	9	3	6
General	7	9	6
Orthopedics	5	3	2
Pain Medicine	3	3	2
Sports Medicine & Rehab	7	18	1
Regenerative Medicine	2	2	1
Surgery	5	5	1
Other	6	3	6
Unknown	1	0	N/A
**Total Number of Board Certifications**	**50**	**53**	**34**
Total Number of Doctors	71	108	

minimally manipulated preparations typically used by the businesses under investigation), (3) using different cell preparation methods compared with the clinic, (4) using a different type of stem cell compared with the clinic, (5) reporting on a medical condition not treated by the clinic, (6) not including a control group in the study, and (7) not reporting that the SCBI was more effective than the control at treating the condition under investigation. Approximately half the papers (n = 126, 48%) met one of these criteria; the other half (n = 135 papers) fell into multiple of the listed criteria.

Medical qualifications of care providers

Stem cell businesses typically listed the medical qualifications of their care providers (85%, n = 50, Figure 1). The biographies of care providers frequently mentioned board certifications and membership of professional associations (Figure 3A). Other markers of credibility included mentioning awards and making it onto magazine lists of "top doctors."

Approximately 70% of the businesses characterized listed at least one board-certified doctor. About 50% of the care providers listed on the websites of sole-focus clinics were board-certified, and 34% in the main-focus clinics. The medical specialties of the board certifications were very varied, with the highest number of care providers certified in specialties relating to sports medicine and rehabilitation, general medicine, and cosmetic practices (Figure 3B).

A total of 34 different medical boards were represented among the care providers across the 59 businesses characterized. Of these 34 boards, 13 are represented by the American Board of Medical Specialties (ABMS), three by the American Osteopathic Association (AOA), and the remaining 18 are independent medical boards (Table S2). Notably, all 25 care providers board-certified in sports medicine and rehabilitation listed certification by the American Board of Physical Medicine and Rehabilitation (a member board of ABMS). In contrast, the 10 care providers listing board certification in alternative medicine were certified by nine different medical boards. Another four care providers listed

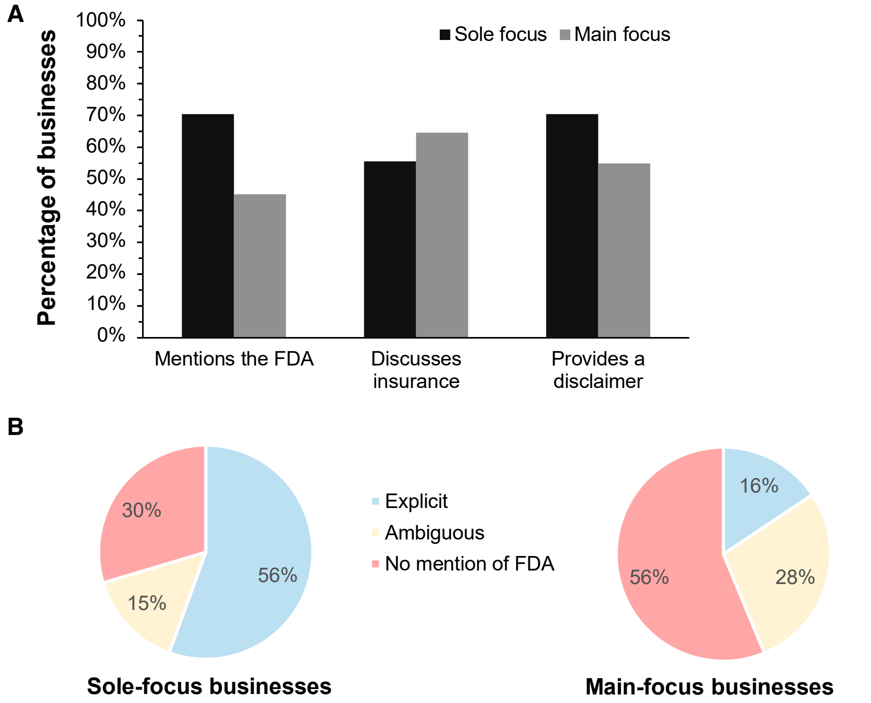

Figure 4. Transparency of stem cell businesses with respect to the experimental status of SCBIs

(A) Percentage of businesses making statements regarding the regulatory, insurance, and evidentiary status of SCBIs.

(B) Type of language used in invoking the FDA by sole-focus (n = 27) and main-focus clinics (n = 32). Examples and specific quotations showing the types of statements classified as "explicit" (blue) or "ambiguous" (yellow) with respect to the regulatory status of SCBIs are provided in Table S5.

board certifications in regenerative medicine, all certified by the American Board of Anti-Aging and Regenerative Medicine (an independent board established in 1992).

Similar heterogeneity was observed with respect to the professional associations listed by care providers (Tables S3 and S4). Over 80 different professional associations were listed by care providers, with associations connected to general medicine and alternative medicine being most represented. In total, seven care providers listed affiliations with two organizations representing regenerative medicine: the American Academy of Anti-Aging Medicine, and the International Federation for Adipose Therapeutics and Science.

Regulatory status of SCBIs

Just over half of the stem cell businesses mentioned the Food and Drug Administration (FDA) on their websites (n = 33). Mention of the FDA was more prevalent on the websites of sole-focus businesses (n = 19, 70%) than main-focus ones (n = 14, 45%) (Figure 4A). Similar proportions provided disclaimers on their website regarding the regulatory status of SCBIs (n = 19 sole-focus and n = 17 main-focus businesses). These disclaimers avoided using language regarding the safety of SCBIs, and explicitly distanced the clinics from any claims about efficacy or the curative potential of SCBIs.

Mentions of the FDA were not uniformly about clarifying the unregulated status of SCBIs (Figure 4B). For sole-focus businesses, 56% (n = 15) included explicit statements that the interventions they offered had not been FDA-approved. But 15% (n = 4) made statements invoking the FDA that could be construed as misleading if a reader was not fully aware of the unregulated status of SCBIs. For main-focus businesses, 16% (n = 5) made explicit statements about the unregulated status of their interventions, and the proportion making potentially misleading statements increased to almost 30% (n = 9). These statements took multiple forms, as detailed in Table S5. For example, a business might suggest that their interventions do not require FDA clearance, or claim they are using FDA-registered facilities and devices, without explicitly mentioning the regulatory status of SCBIs. In two cases, businesses made explicit claims that their SCBIs had been FDA-approved.

DISCUSSION

Our study of direct-to-consumer stem cell businesses in the Southwest United States offers detailed insight into the forms of evidence mobilized by these clinics in promoting their services. In 2016, Turner and Knoepfler identified that the Southwest states captured approximately one-third of the SCBI marketplace in the United States, and included four of the seven "hot-spot" cities (Beverly Hills, Los Angeles, Phoenix, and Scottsdale). While our study reports on a relatively small and geographically contained sample,

we suggest the forms of evidence we identified are represented on the websites of direct-to-consumer stem cell clinics across the Southwest and the United States more generally, and that similar patterns in frequency of use are likely. Several of the forms of evidence we identify have also been observed on the websites of stem cell clinics in Australia and Japan (Munsie et al., 2017), but with little documentation regarding their frequency of use.

We identify that stem cell businesses do not rely extensively on gold-standard forms of scientific evidence like randomized clinical trials, nor do they frequently cite patented products or procedures in support of their services. Rather, we observe that they rely extensively on forms of evidence that we describe as scientifically or medically ambiguous. By ambiguous evidence, we mean that there is a scientific or medical basis to the information being mobilized, but the interpretation of this evidence is highly context-dependent. Forms of ambiguous evidence mobilized by DTC clinics include (1) highlighting the training and expertise of the clinic care providers, (2) relying heavily on the use of scientific and technical jargon, (3) citing peer-reviewed scientific research papers, and (4) invoking patient testimonials. We use the term ambiguous to draw attention to the gray areas that are made visible when these forms of evidence are put under scrutiny by researchers and/or prospective consumers. This term also highlights the responsibility placed on individuals to interpret the evidence. We briefly discuss each of these gray areas below.

Training and expertise of care providers

Surveys show that the public views board certification as an important consideration when choosing a physician (Gallup, 2003; Freed et al., 2010). However, board certification alone does not mean a physician is qualified to perform regenerative medicine treatments; the specific nature of their qualifications must be considered. There are currently no ABMS or AOA medical boards focused on cellular or regenerative medicine. Furthermore, the heterogeneity in medical boards (>30 boards) represented by the care providers working at the sampled businesses suggests there is currently little consistency in training and oversight of the regenerative medicine interventions offered by these clinics. While board certification is nominally a marker of clinical expertise, experimental and unregulated interventions pose more of a challenge for prospective patients in determining whether the specific credentials of a care provider are appropriate to the treatment they are seeking.

Textbook-style jargon

Textbooks present authoritative views about the current state of knowledge in science and medicine, without necessarily relying on detailed referencing to support each fact or point. Critical to the authority of a textbook is the credibility of the author(s), with core textbooks in science and medicine typically authored by leading, trusted figures in their respective fields. The technical descriptions of stem cells, regenerative medicine, and treatment options detailed on almost all the DTC clinic websites analyzed in this study are written in "textbook" style, but it is not clear exactly who the authors are and what authority or expertise they bring to the table. As such, evaluating the accuracy of the claims made becomes more challenging.

Scientific research papers

About one-third of the DTC stem cell clinics analyzed here cite one or more scientific research papers in support of the services they offer. These articles are published in peer-reviewed, credible scientific journals, and so at face value can be understood to present sound scientific findings. The key challenge for prospective patients is in interpreting whether the specific details of a cited study lend support for the particular treatment they are seeking. Our analysis identifies that this is very rarely the case, with fewer than 2% of the cited papers reporting clinical improvements in humans using exactly the same kind of same-day, minimally manipulated cell preparations offered by DTC clinics. Prospective patients are left to interpret the relevance of any differences between findings of published studies and the specific treatment they are considering.

Patient testimonials

There is a long history in medicine of publishing case reports that focus on a single individual. Acknowledging that patient testimonials are clearly not equivalent to case reports, we nonetheless identify that there is some tolerance within the medical profession for sample sizes comprising one individual. A number of case reports have been published in recent years highlighting adverse effects faced by small numbers of individuals in response to unregulated SCBIs (Dlouhy et al., 2014; Dobke et al., 2013; Kuriyan et al., 2017). The content of medical case reports is very different from patient testimonials, with the latter being almost exclusively cherry-picked by a business to reflect well upon its practice and its care providers (if indeed the testimonials are provided by real patients of the clinic). These testimonials may be persuasive to potential customers, and may indeed report on genuine clinical improvement experienced by an individual; however, they cannot offer certainty regarding the cause of improvement – an unresolvable ambiguity that a prospective patient must contend with in interpreting such testimonials.

Conclusions

Direct-to-consumer stem cell businesses use many forms of evidence in marketing SCBIs online. The findings from this study highlight the significant interpretive responsibility placed on individuals when it comes to evaluating the credibility of a given business – they would ideally attend to the type of evidence and its specific content, as well as the context in which it is presented. For prospective patients seeking to do their due diligence, our analysis highlights the following questions:

- Are the qualifications of the care providers at the clinic appropriate for the specific treatment being sought? What is the history and credibility of a given medical board in certifying practitioners?
- Do the research studies cited on the clinic's website provide direct and unambiguous support for the specific treatment being sought?
- Is the clinic explicit about the current regulatory status of experimental SCBIs?

We see roles for professional societies and regulatory agencies in helping to navigate these questions. Medical boards can make explicit statements about their position with respect to SCBIs. In our sample, the American Board of Physical Medicine and Rehabilitation (ABPMR) stands out among the 30+ medical boards listed by clinic practitioners, with 25% of the board-certified practitioners citing certification by the ABPMR. Understanding whether the ABPMR offers explicit training in SCBIs or regenerative medicine, and what its position is with respect to such treatments, could be informative for prospective patients in evaluating the expertise of physicians certified by this board.

Professional societies like the International Society for Stem Cell Research (ISSCR) can offer detailed guidance on factors to consider when reading scientific journal articles about stem cells; our analysis identifies several variables to include in such guidance. This list might also be relevant to the Federal Trade Commission (FTC) for their role in protecting consumers from deceptive marketing practices around SCBIs (FTC, 2018), as it provides criteria against which to evaluate whether published materials offer a clear evidence base for specific SCBIs. We identify that most clinics citing peer-reviewed scientific literature on their websites are not citing studies that report on the specific interventions they offer, calling into question the evidence base of their marketing claims. Furthermore, about half the clinics in our sample do not make explicit statements about the unregulated status of the SCBIs they offer; this might also be a productive area of focus for the FTC.

Finally, while these are steps that can be taken by the scientific and medical establishment to restrict deceptive practices and assist prospective patients with interpreting evidence, we note the limitations of such efforts for scientists, medical practitioners, and regulators concerned about the very existence of the direct-to-consumer SCBI marketplace. Making prospective patients aware of the "unscientific" or less scientifically legitimate nature of particular forms of evidence is unlikely to eliminate demand for SCBIs (Brown, 2009). Our empirical findings showing the multiple forms of evidence presented by stem cell clinics lend support to work describing direct-to-consumer SCBIs as straddling an increasingly blurred line between medicine and "biomedical 'lifestyle' products" (Erikainen et al., 2020; Saukko et al., 2010). The socio-political context in which this marketplace has emerged is one in which individuals are increasingly framed as consumers rather than patients, and are expected to take active roles in promoting and advocating for their own health (Erikainen et al., 2020). Many of the businesses characterized here use language that positions the prospective consumer as being in charge of their own health and challenges traditional sources of expert advice. Our study offers a baseline characterization of the frequency and types of evidence used by DTC stem cell clinics. We call for more empirical work to understand the priorities and logics used by prospective consumers when considering experimental SCBIs (Petersen et al., 2013; Rachul, 2011; Waldby et al., 2020), and specifically how they approach and interpret the many forms of scientifically and medically ambiguous evidence that currently exist in the SCBI marketplace.

EXPERIMENTAL PROCEDURES

We gathered publicly available material from the websites of stem cell businesses, focusing on forms of evidence used in marketing their services, as well as any statements regarding the regulatory status of the SCBIs on offer. Data collection was performed between late July and early September 2020. We began with the 169 businesses characterized in Frow et al. (2019), which used Internet search terms including the six Southwestern states (Arizona, California, Colorado, Nevada, New Mexico, Utah) and large cities in the Southwest United States to add 41 businesses to the dataset of 128 Southwest clinics published by Turner and Knoepfler in 2016. We then narrowed down this set of 169 businesses to include only those with a "sole focus" or a "main focus" on offering SCBIs; we excluded businesses for which SCBIs were listed as an offering but were not a visible focus of the clinic's practice, so that the types of evidence presented on clinic websites could be clearly associated with SCBIs. The total number of businesses characterized for this study was 59 (27 sole focus, 32 main focus). This number is lower than the total number of sole-focus (n = 42) and main-focus businesses (n = 65) identified in Frow et al. (2019) owing to clinic turnover (see Supplemental Experimental Procedures for details).

An overview of the data collected for each business is presented, in de-identified form, in Supplemental Table S1. There is great heterogeneity in the terminology and type of information presented on the websites of stem cell businesses. Several broader categories were determined during the process of qualitative data analysis; the categorizations used for care provider specialties and professional affiliations are provided in Tables S2 and S4. Additional details about data analysis methods are provided in the Supplemental Experimental Procedures.

SUPPLEMENTAL INFORMATION

Supplemental information can be found online at https://doi.org/10.1016/j.stemcr.2021.10.007.

AUTHOR CONTRIBUTIONS

E.K.F. and D.A.B. conceptualized the project. E.K.F. supervised the project. M.C., A.R., and E.K.F. were study investigators. M.C., A.R., and E.K.F. provided data curation and analysis.; M.C., A.R., and E.K.F. wrote the original draft. D.A.B and E.K.F. reviewed and edited the manuscript. M.C., A.R., and E.K.F. visualized the project.

CONFLICTS OF INTEREST

The authors declare no competing interests.

ACKNOWLEDGMENTS

E.K.F. acknowledges funding from the Lincoln Center for Applied Ethics at Arizona State University. The authors thank Haley Nadone and Bryson Becker for preliminary work on board certifications and FDA-related language, respectively.

Received: May 6, 2021
Revised: October 12, 2021
Accepted: October 13, 2021
Published: November 11, 2021

REFERENCES

Brown, S. (2009). The new deficit model. Nat. Nanotechnol. *4*, 609–611.

Dlouhy, B., Awe, O., Rao, R.C., Kirby, P.A., and Hitchon, P.W. (2014). Autograft-derived spinal cord mass following olfactory mucosal cell transplantation in a spinal cord injury patient: case report. J. Neurosurg. Spine *21*, 618–622.

Dobke, M., Bailey, J., Bhavsar, D., Saba, S., and Mailey, B. (2013). Necrotizing metachronous facial ulcerations after "stem cell face lift". Ann. Plast. Surg. *70*, 392–396.

Erikainen, S., Couturier, A., and Chan, S. (2020). Marketing experimental stem cell therapies in the UK: biomedical lifestyle products and the promise of regenerative medicine in the digital era. Sci. as Cult. *29*, 219–244.

Federal Trade Commission; Regenerative Medical Group Inc.; Telehealth Medical Group Inc (2018). United States District Court for Central District of California, *8*, 18-cv-01838.

Freed, G.L., Dunham, K.M., Clark, S.J., and Davis, M.M. (2010). Research Advisory Committee of the American Board of Pediatrics: perspectives and preferences among the general public regarding physician selection and board certification. J. Pediatr. *156*, 841–845.

Frow, E.K., Brafman, D.A., Muldoon, A., Krum, L., Williams, P., Becker, B., Nelson, J.P., and Pritchett, A. (2019). Characterizing direct-to-consumer stem cell businesses in the Southwest United States. Stem Cell Rep. *13*, 1–7.

Fu, W., Smith, C., Turner, L., Fojtik, J., Pacyna, J.E., and Master, Z. (2019). Characteristics and scope of training of clinicians participating in the US direct-to-consumer market for unproven stem cell interventions. JAMA *321*, 2463.

Gallup Organization for the American Board of Internal Medicine (2003). Awareness of and Attitudes toward Board-Certification of Physicians (The Gallup Organization).

ISSCR, 2015. Nine things to know about stem cell treatments. https://www.closerlookatstemcells.org/stem-cells-medicine/nine-things-to-know-about-stem-cell-treatments/.

Knoepfler, P.S. (2017). The stem cell hard sell: report from a clinic's patient recruitment seminar. Stem Cells Transl. Med. *6*, 14–16.

Knoepfler, P.S., and Turner, L.G. (2018). The FDA and the US direct-to-consumer marketplace for stem cell interventions: a temporal analysis. Regen. Med. *13*, 19–27.

Knoepfler, P. (2020). Stem cell journals impact factors, editors & experiences, The Niche. https://ipscell.com/2020/06/helpful-2020-list-of-stem-cell-journals/.

Kuriyan, A.E., Albini, T.A., Townsend, J.H., Rodriguez, M., Pandya, H.K., Leonard, R.E., Parrott, B., Rosenfeld, P.J., Flynn, H.W., and Goldberg, J.L. (2017). Vision loss after intravitreal injection of autologous "stem cells" for AMD. N. Engl. J. Med. *376*, 1047–1053.

Munsie, M., Lysaght, T., Hendl, T., Tan, H.-Y.L., Kerridge, I., and Stewart, C. (2017). Open for business: a comparative study of websites selling autologous stem cells in Australia and Japan. Regen. Med. *12*, 777–790.

Petersen, A., Seear, K., and Munsie, M. (2013). Therapeutic journeys: the hopeful travails of stem cell tourists. Soc. Health Illn. *36*, 670–685.

Rachul, C. (2011). What have I got to lose?': an analysis of stem cell therapy patients' blogs. Health Law Rev *21*, 5–12.

Rachul, C.M., Percec, I., and Caulfield, T. (2015). The fountain of stem cell-based youth? Online portrayals of anti-aging stem cell technologies. Aesthet Surg J *35*, 730–736.

Saukko, P.M., Reed, M., Britten, N., and Hogarth, S. (2010). Negotiating the boundary between medicine and consumer culture: online marketing of nutrigenetic tests. Soc. Sci. Med. *70*, 744–773.

Sipp, D., Caulfield, T., Kaye, J., Barfoot, J., Blackburn, C., Chan, S., De Luca, M., Kent, A., McCabe, C., Munsie, M., et al. (2017). Marketing of unproven stem cell-based interventions: a call to action. Sci. Trans. Med. *9*, eaag0426.

Turner, L., and Knoepfler, P. (2016). Selling stem cells in the USA: assessing the direct-to-consumer industry. Cell Stem Cell *19*, 154–157.

Turner, L. (2018). The US direct-to-consumer marketplace for autologous stem cell interventions. Perspect. Biol. Med. *61*, 7–24.

Waldby, C., Hendl, T., Kerridge, I., Lipworth, W., Lysaght, T., Munsie, M., and Stewart, C. (2020). The direct-to-consumer market for stem cell-based interventions in Australia: exploring the experiences of patients. Future Med. *15*, 1238–1249. https://doi.org/10.2217/rme-2019-0089.

Zarzeczny, A., Atkins, H., Illes, J., Kimmelman, J., Master, Z., Robillard, J.M., Snyder, J., Turner, L., Zettler, P.J., and Caulfield, T. (2018). The stem cell market and policy options: a call for clarity. J. Law Biosci. *5*, 743–758.

Stem Cell Reports, Volume *16*

Supplemental Information

Weighing up the evidence used by direct-to-consumer stem cell businesses

Margaret Cook, Alexandra Richey, David A. Brafman, and Emma K. Frow

Supplemental Information

Table S1. Data summary for direct-to-consumer stem cell businesses in the Southwest US (related to Experimental Procedures). State locations are provided for each business, but specific business names are not identified.

See Excel file for Table S1.

Table S2. List of all medical boards cited by care providers across the businesses characterized (*n*=179 care providers across 59 businesses), grouped by medical specialty. Boards represented by the American Board of Medical Specialties (ABMS) and the American Osteopathic Association (AOA) are noted. Related to **Fig. 3**.

Categories	Medical Boards Contained in Category	ABMS or AOA
Cosmetic	American Board of Plastic Surgery	ABMS
	American Board of Cosmetic Surgery	
	American Board of Facial Plastic and Reconstructive Surgery	
	American Academy of Aesthetic Medicine	
	American Board of Hair Restoration Surgery	
	American Board of Dermatology	ABMS
General	American Board of Family Medicine	ABMS
	The American Board of Internal Medicine	ABMS
	American Board of Preventive Medicine in Occupational Medicine	ABMS
	National Commission on Certification of Physician Assistants	
	American Academy of Nurse Practitioners	
	American Association of Medical Assistants	
Pain Medicine	American Board of Pain Medicine	
	American Board of Interventional Pain Physicians	
Alternative	American Osteopathic Academy of Sports Medicine	
	American Osteopathic Board of Physical Medicine and Rehabilitation	ABMS
	American Board of Holistic Medicine	
	National Board of Osteopathic Medical Examiners	
	American Osteopathic Board of Family Practice	AOA
	American Board of Functional Medicine	
	American Osteopathic Board of Family Physicians	AOA
	American Osteopathic Board of Neuromusculoskeletal Medicine	AOA
	National Certification Commission for Acupuncture and Oriental Medicine	
Orthopedics	American Board of Orthopaedic Surgery	ABMS
	American Board of Spine Surgery	

Sports Medicine & Rehab	American Board of Physical Medicine and Rehabilitation	ABMS
Surgery	American Board of Anesthesiology	ABMS
Regenerative Medicine	American Board of Anti-Aging & Regenerative Medicine	
Other	The American Board of Otolaryngology	ABMS
	American Board of Psychiatry and Neurology	ABMS
	American Board of Radiology	ABMS
	American Board of Urology	ABMS
	American College of Thermography	
	American Board of Addiction Medicine	

Table S3. Professional associations listed by all care providers on the business websites studied (*n*=179 care providers across 59 businesses). Professional associations were grouped into nine categories; a complete list of associations is available in Table S4.

**Medical Specialty**	**Professional associations at sole focus businesses (# of care providers)**	**Professional associations at main focus businsses (# of care providers)**	**Number of professional associations represented**
Alternative Medicine	2	14	13
Cosmetic	10	4	9
General	20	4	19
Orthopedics	9	15	9
Pain Medicine	1	8	6
Sports Medicine & Rehab	0	5	3
Regenerative Medicine	4	3	2
Surgery	8	5	7
Other	8	7	14
**Total Number of Professional Associations**	**76**	**65**	**82**
Number of Doctors listing Professional Associations	25	25	
Total Number of Doctors	71	108	

Table S4. List of all professional associations cited by care providers across the businesses characterized, grouped by medical specialty as in Table S3.

See Excel file for Table S4.

Table S5. Type of statement made in invoking the FDA, broken down by sole-focus (*n*=27) and main-focus clinics (*n*=32). Specific quotations illustrate the types of statements classified as "explicitly" acknowledging the unregulated status of SCBIs (blue) or "ambiguous" with respect to the regulatory status of SCBIs (yellow). Color-coding matches the pie charts in **Fig. 4B**.

**Categorization of FDA Statement**	**Total # of businesses**	**# of sole-focus businesses**	**# of main-focus businesses**	**Example statement**
Explicit statement that stem cell treatments are not FDA-approved	**13**	8	5	"Regenerative medicine (RM) is a fast-growing, highly-developed technology that is increasingly being used to manage conditions such as chronic and acute pain. The FDA has not yet approved the use of regenerative treatments and medicine to remedy every pain condition and case. However, regenerative treatments and medicine might be appropriate for you, depending on your specific case."
Explicit statement that stem cell treatments are not FDA-approved but suggesting compliance with 21 CFR Part 1271	**7**	7	0	Is our procedure FDA-approved?<br>"NO. However, [our] surgical procedures fall under the category of physician's practice of medicine, wherein the physician and patient are free to consider their chosen course of treatment. The FDA does have guidelines about treatment and manipulation of a patient's own tissues. At [company] we meet these guidelines by providing same day treatment with the patient's own cells that undergo no manipulation and are inserted during the same procedure."
Explicit statement that stem cell treatments are not FDA-approved but suggesting these treatments do not require FDA approval/clearance	**1**	1	0	"This procedure is not FDA approved and cannot be FDA approved because it does not involve anything other than a person's own tissue. The FDA does not approve or disapprove of any procedures like this, because it is a type of operation that they have no domain over. Instead, this is a procedure in which the person's own cells are transferred in a concentrated form into an area where he or she may need them."
Statement claiming compliance with the FDA regulations set forth in 21 C.F.R. Part 1271 while also suggesting that stem cell treatments do not require FDA approval/clearance	**2**	1	1	"This is a medical procedure; the FDA does not "approve" or "disapprove" medical procedures (like gall bladder surgery for example). Medical procedures are performed at the discretion of the practicing physician and their state's board. All of [Company's] Stem Cell and Blood Platelet Procedures performed in the U.S. today are same-day procedures that are compliant with CFR 21 Part 1271, falling under the same surgery exemption discussed in 1271.15 (b)."
Statement invoking an FDA-registered clinic/facility	**2**	2	0	"We work with [other company], an FDA-approved stem cell provider. Every sample we receive is microbiology tested and registered with the FDA, Tissue Bank and NIH & FWA."

Statement invoking an FDA-registered device	**1**	0	1	"Our medical director and founding surgeon, [Doctor], offers our hair loss patients the most sophisticated FDA approved stem cell device for obtaining adult stem cells from the fat cells of patients."
Suggesting broad compliance with FDA	**5**	0	5	"[Business] follows all FDA guidance related to stem cell therapy. You are encouraged to consult with your primary care physician prior to undergoing a stem cell therapy."
Statement claiming that stem cell treatments are FDA approved/cleared	**2**	0	2	"[Business] offers the only FDA approved autologous adipose stem cell regeneration system. The potential and future of the use of stem cells in aiding recovery of multiple soft tissue injuries is now offered in [region]!"
Do not mention the FDA	**25**	8	18	N/A

Supplemental Experimental Procedures

Online data collection

DTC stem cell businesses were characterized between late July and early September 2020. Information was collected from the public sections of business websites and collated in an Excel spreadsheet. For each characterized business, screenshots were taken of each webpage presenting information that could be interpreted as evidence supporting the SCBIs on offer at the clinic (using Snagit software), to construct an archive for future reference. The data collected reflects the public presentation of a stem cell business; it is assumed to be accurate, but is not guaranteed to represent the actual practices of a given business. For example, a given clinic might not list all the conditions they treat using SCBIs, or might not in practice treat all the conditions they list online.

Peer-reviewed scientific papers. After identifying the subset of clinics citing peer-reviewed scientific papers, citations for each article were obtained and the corresponding articles found through PubMed. A total of 261 journal articles were identified. The abstract for each paper was read, and a set of categories was iteratively developed by the authors (A.R. and E.K.F.) to evaluate each paper in relation to the SCBIs on offer at the business citing the paper. The following categories were used: (1) whether the paper was investigating a specific stem-cell-based treatment, (2) what condition was being tested or treated using stem cells, (3) whether the research subjects were humans or animals, (4) the sources of stem cells used in the paper, (5) whether the cells in the preparation had been cultured or manipulated, (6) the size of the study performed, and (7) whether or not the study showed that stem cell treatment resulted in positive clinical outcomes compared to the control group. This information was cross-referenced with the medical conditions treated by the business citing the paper, together with the treatment method(s) presented by the business and the source(s) of stem cells they claimed to use. Based on this information, a determination was made for each cited paper about whether it supported the SCBIs offered by that business.

Among the businesses evaluated, three larger franchises were represented: Regenexx, R3 and the Cell Surgical Network (CSN). CSN and Regenexx have both published research papers by their lead physicians and researchers; these were analyzed as described above. R3 also referenced peer-reviewed articles in their informational videos, but these were not authored by individuals affiliated with R3. These were also analyzed as described above. CSN also hosts a large database of regenerative medicine research on their website. The purpose of this database is stated as

giving physicians and researchers "the opportunity to share not only their data, but their vital clinical experiences [in order to help] each other to achieve higher levels of scientific understanding and [optimize] medical care."[1] This database covers many forms of regenerative medicine and has entries discussing research for over 30 medical conditions in the fields of urology, cardiology and pulmonology, orthopedics, neurology, autoimmune diseases, and ophthalmology. The entries in the database include peer-reviewed journal articles covering stem-cell-based and non-stem-cell-based treatments, review articles, and information on ongoing clinical trials. Since this extensive database is stated as being primarily for the benefit of physicians and researchers (not patients considering SCBIs), these entries were not individually evaluated.

Medical Specialties. The care providers listed for each stem cell business were copied into the data collection spreadsheets, together with any specialty and/or board certification information listed on the business website (**Supplemental Table S2**), and any professional associations or memberships listed (**Supplemental Table S4**). Once identified, board certifications and professional affiliations were grouped into 9 categories for analysis (**Fig. 3B** and **Supplemental Table S3**).

Rate of clinic turnover. Since the publication of Frow et al (2019), we note that approximately 33% of the sole-focus businesses (14 out of 42) no longer had a web presence or no longer offered SCBIs, and that a similar proportion of the main-focus businesses (24 out of 65) no longer had a web presence or no longer offered stem-cell-based or regenerative medicine interventions. This is higher than the 10% turnover noted by Wu et al. (2019) in their study of stem cell clinics in California, Florida and Texas between 2016-2018. These numbers suggest a highly dynamic market for SCBIs. We speculate that the higher turnover might reflect the formalization of FDA guidance regarding cell- and tissue-based products in November 2017, with the grace period for compliance to expire in May 2021. Some businesses had also shifted their emphasis with respect to SCBIs since Frow et al. (2019), with 6 out of 42 businesses moving away from a sole focus on SCBIs to offering a greater variety of services. Conversely, 6 out of 65 of the 'main-focus' businesses previously characterized had narrowed their focus exclusively to SCBIs.

[1] See https://stemcellrevolution.com/about-us/our-mission/.

References

Fu, W., Smith, C., Turner, L., Fojtik, J., Pacyna, J.E., and Master, Z. (2019) Characteristics and scope of training of clinicians participating in the US direct-to-consumer market for unproven stem cell interventions. JAMA 321(24), 2463-2463.
